# Crystal structure of *N*-acetylmannosamine kinase from *Fusobacterium nucleatum*

**DOI:** 10.1107/S2053230X17007439

**Published:** 2017-05-31

**Authors:** Rhawnie Caing-Carlsson, Parveen Goyal, Amit Sharma, Swagatha Ghosh, Thanuja Gangi Setty, Rachel A. North, Rosmarie Friemann, S. Ramaswamy

**Affiliations:** a Department of Chemistry and Molecular Biology, University of Gothenburg, Box 462, 40530 Gothenburg, Sweden; b Centre for Antibiotic Resistance Research (CARe), University of Gothenburg, Box 440, 40530 Gothenburg, Sweden; c Atomic Physics, Department of Physics, Lund University, Professorsgatan 1, 22363 Lund, Sweden; d Institute for Stem Cell Biology and Regenerative Medicine, GKVK Post, Bangalore 560 065, India; e School of Life Sciences, TransDisciplinary University, Bangalore 560 064, India; f Biomolecular Interaction Centre and School of Biological Sciences, University of Canterbury, Private Bag 4800, Christchurch 8041, New Zealand; g Department of Structural Biology, Stanford University School of Medicine, 299 Campus Drive West, Stanford, CA 94305-5126, USA

**Keywords:** sialic acid catabolism, *N*-acetylmannosamine kinase, *Fusobacterium nucleatum*

## Abstract

Sialic acids comprise a varied group of nine-carbon amino sugars that are widely distributed among mammals and higher metazoans. Some human commensals and bacterial pathogens can scavenge sialic acids from their environment and degrade them for use as a carbon and nitrogen source. The enzyme *N*-acetylmannosamine kinase (NanK; EC 2.7.1.60) belongs to the transcriptional repressors, uncharacterized open reading frames and sugar kinases (ROK) superfamily. NanK catalyzes the second step of the sialic acid catabolic pathway, transferring a phosphate group from adenosine 5′-triphos­phate to the C6 position of *N*-acetylmannosamine to generate *N*-acetylmannosamine 6-phosphate. The structure of NanK from *Fusobacterium nucleatum* was determined to 2.23 Å resolution by X-ray crystallography. Unlike other NanK enzymes and ROK family members, *F. nucleatum* NanK does not have a conserved zinc-binding site. In spite of the absence of the zinc-binding site, all of the major structural features of enzymatic activity are conserved.

## Abstract 2

The enzyme *N*-acetylmannosamine kinase (NanK) catalyzes the second step of the bacterial sialic acid catabolic pathway. Here, the structure of *F. nucleatum* NanK is presented at 2.23 Å resolution.

## 1. Introduction   

**Figure 1:**
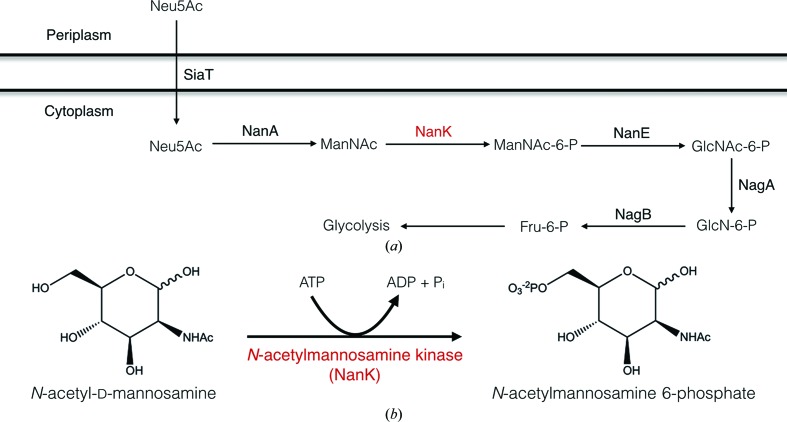
(*a*) Sialic acid catabolism in *F. nucleatum*. SiaT, transporter; NanA, lyase; NanK, kinase; NanE, epimerase; NagA, deacetylase; NagB, deaminase. (*b*) The chemical reaction catalyzed by *N*-acetylmannosamine kinase.

Sialic acids comprise a large family of acidic sugars that contain a core nine-carbon backbone (Angata & Varki, 2002 ▸; Vimr *et al.*, 2004 ▸; Varki, 1992 ▸). The most prevalent type of sialic acid is *N*-acetylneuraminic acid (Neu5Ac), which is found at the terminal positions of glycoconjugates in humans and other deuterostomes. Sialylation of cell surfaces is crucial for cell–cell interactions and for a range of biological functions that involve cell-signalling processes and modulation of the immune response (Tanner, 2005 ▸; Varki, 2007 ▸; Vimr *et al.*, 2004 ▸; Kazatchkine *et al.*, 1979 ▸; Lanoue *et al.*, 2002 ▸). Prompted by evolution to adapt to the sialic acid-rich milieu, many bacteria have developed mechanisms to competitively secure their niche on mucosal surfaces (Almagro-Moreno & Boyd, 2009*b* ▸). Bacteria acquire sialic acids either by cleaving them from the host’s glycoconjugates or by scavenging (Vimr, 2013 ▸). Once the sialic acid has been transported into the cytosol by specific transporters, some bacteria can incorporate it as a non­reducing terminal sugar on their cell surface for molecular mimicry, and thereby evade the host’s innate and adaptive immune response, or can degrade it for use as a carbon and nitrogen source (Mulligan *et al.*, 2011 ▸). The ability to utilize sialic acid as an energy source is chiefly exploited by commensal and pathogenic bacteria and requires a cluster of genes, known as the *nan*–*nag* cluster (Almagro-Moreno & Boyd, 2009*b* ▸, 2010 ▸; Haines-Menges *et al.*, 2015 ▸; Fig. 1 ▸). The sialic acid *nan*–*nag* gene cluster was identified in the genome of a *Fusobacterium* species which exists as a commensal in the gastrointestinal tract and as a periodontal pathogen (Almagro-Moreno & Boyd, 2009*a* ▸). Once host sialic acid has been transported across the cytoplasmic membrane (by a two-component sialic acid tripartite ATP-independent periplasmic transport system in *F. nucleatum*), degradation of Neu5Ac to fructose 6-phosphate starts with the conversion of sialic acid by an *N*-acetylneuraminate lyase (NanA), yielding *N*-acetyl­mannosamine (ManNAc) and pyruvate. A phosphoryl group from ATP is then transferred to ManNAc by a kinase (NanK), producing *N*-acetyl­mannosamine 6-phosphate (ManNAc-6-P), which in turn is converted to *N*-acetyl­glucosamine 6-phosphate (GlcNAc-6-P) by *N*-acetyl­mannosamine-6-phosphate 2-epi­merase. Finally, GlcNAC-6-P is deacylated by *N*-acetyl­glucosamine-6-phosphate deacetylase (NagA) and is subsequently deaminated by glucosamine-6-phosphate deaminase (NagB) to yield fructose 6-phosphate (Vimr & Troy, 1985 ▸).

*F. nucleatum* *N*-acetylmannosamine kinase (FnNanK) belongs to the repressor, open reading frame, kinase (ROK) superfamily of proteins. This collection of polypeptides is primarily composed of transcriptional repressors, sugar kinases and other unknown gene clusters (Titgemeyer *et al.*, 1994 ▸). The salient unifying features of the ROK scaffold are a nucleotide-binding region in the N-terminal region, a strictly conserved catalytic aspartate residue that serves as a Schiff base during phosphoryl transfer and a zinc-binding motif that is implicated in the stability of the active site of the enzyme (Martinez *et al.*, 2012 ▸; Conejo *et al.*, 2010 ▸). Structural representatives of human *N*-acetylmannosamine kinase (hMNK) domain of UDP-*N*-acetylglucosamine-2-epimerase/*N*-acetyl­mannosamine kinase (Tong *et al.*, 2009 ▸; Martinez *et al.*, 2012 ▸) and two putative NanKs from *Escherichia coli* (EcNanK; PDB entry 2aa4; New York SGX Research Center for Structural Genomics, unpublished work) and *Listeria monocytogenes* (LmNanK; PDB entry 4htl; Midwest Center for Structural Genomics, unpublished work) have been deposited in the Protein Data Bank. The overall fold is a butterfly-shaped homodimer. Each monomer consists of two domains that are connected by two hinge loops, allowing the kinase to change from an open conformation to a closed conformation upon substrate binding.

Here, we present the structural analysis of *N*-acetyl­mannosamine kinase from *F. nucleatum*. This structure is important for inhibitor design, which may lead to the development of antimicrobial agents for the treatment of periodontal disease.

## 2. Materials and methods   

### 2.1. *F. nucleatum* NanK production   

The gene encoding *F. nucleatum* NanK was synthetically generated (GeneArt) and cloned into a pET300 NT/DEST expression vector containing an N-terminal His tag. The recombinant protein was expressed in *E. coli* BL21(DE3) cells (Novagen). The cells were grown at 37°C in Luria broth (LB) medium supplemented with 100 µg ml^−1^ ampicillin until they reached mid-log phase (OD_600_ = ∼0.5–0.7). The cells were induced with 0.2 m*M* isopropyl β-d-1-thiogalactopyranoside (IPTG) and were grown at 20°C for a further 18 h. The cells were harvested by centrifugation at 5000*g* for 30 min and were resuspended in buffer *A* [20 m*M* Tris–HCl pH 8.0, 300 m*M* NaCl, 10 m*M* imidazole, 5%(*v*/*v*) glycerol].

The cells were disrupted using an EmulsiFlex-C3 (Avestin) at 124 MPa for two cycles. The cell debris was removed by centrifugation at 107 000*g* for 30 min at 4°C. Macromolecule-production information is summarized in Table 1 ▸.

### 2.2. Protein purification   

FnNanK was purified by affinity chromatography at 4°C using a 5 ml HisTrap FF column (GE Healthcare) pre-equilibrated with buffer *A*. The bound protein was washed with buffer *A* and the protein was then eluted with buffer *B* [20 m*M* Tris–HCl pH 8.0, 300 m*M* NaCl, 500 m*M* imidazole, 5%(*v*/*v*) glycerol]. As a final polishing step, the protein was loaded onto a HiLoad 16/600 Superdex 200 size-exclusion column (GE Healthcare) pre-equilibrated with buffer *C* [20 m*M* Tris–HCl pH 8.0, 300 m*M* NaCl, 5%(*v*/*v*) glycerol, 1 m*M* DTT]. The purity of the eluted protein samples was evaluated using SDS–PAGE. The pure samples corresponding to FnNanK were pooled together and concentrated using Vivaspin concentrators to a final concentration of 14 mg ml^−1^. The protein concentration was determined using an ND-1000 spectrophotometer at 280 nm, using an extinction coefficient of 27 005 *M* ^−1^ cm^−1^ and a molecular weight of 33.9 kDa.

### 2.3. Crystallization   

The initial screening for crystallization conditions for *F. nucleatum* NanK was performed at 293 K using a Mosquito nanolitre-dispensing robot (TTP Labtech) with Crystal Screen HT (Hampton Research). The sitting-drop vapour-diffusion method was used, mixing 0.2 µl protein solution (14 mg ml^−1^) and 0.2 µl reservoir solution. Within one week, rod-shaped crystals of FnNanK were obtained using a reservoir solution consisting of 0.2 *M* lithium sulfate monohydrate, 0.1 *M* Tris–HCl pH 8.5, 30%(*w*/*v*) PEG 4000. The crystals were flash-cooled in liquid nitrogen prior to the diffraction experiment. Crystallization conditions are summarized in Table 2 ▸.

### 2.4. Data collection and processing   

The crystals of FnNanK diffracted to 2.23 Å resolution. X-ray diffraction data were collected at 100 K on the I911-3 beamline at the MAX-lab National Research Laboratory for Nuclear Physics and Synchrotron Radiation Research, Lund, Sweden using X-rays at a wavelength of 1.0 Å. Diffraction intensities were processed and integrated using *iMosflm* (Battye *et al.*, 2011 ▸) and were scaled using *AIMLESS* from the *CCP*4 program suite (Evans & Murshudov, 2013 ▸). Data-collection and processing statistics are shown in Table 3 ▸.

### 2.5. Structure solution and refinement   

The structure of *F. nucleatum* NanK was determined by molecular replacement using the coordinates of *L. monocytogenes* NanK (PDB entry 4htl) as a search model using *Phaser* (Read, 2001 ▸) within the *PHENIX* software suite (Adams *et al.*, 1999 ▸, 2011 ▸). The *phenix.autobuild* program was used for initial model building and electron-density improvement. Subsequently, *phenix.refine* was used for rigid-body refinement, maximum-likelihood least-squares refinement, simulated annealing and addition of water molecules to the structure. Manual inspection and model building were performed using *Coot* (Emsley *et al.*, 2010 ▸). Structure-solution and refinement statistics are summarized in Table 4 ▸.

## 3. Results and discussion   

### 3.1. Protein production, purification and crystallization   

*F. nucleatum* NanK (FnNanK) was successfully expressed and purified using a two-step procedure consisting of affinity and size-exclusion chromatography and was concentrated to a final concentration of 14 mg ml^−1^. The preparations were homogenous when analyzed by SDS–PAGE and size-exclusion chromatography. Using the sitting-drop vapour-diffusion method, rod-shaped crystals formed within one week using a reservoir solution consisting of 0.2 *M* lithium sulfate monohydrate, 0.1 *M* Tris–HCl pH 8.5, 30%(*w*/*v*) PEG 4000.

### 3.2. Crystal structure of *F. nucleatum* NanK   

The structure of FnNanK has one homodimer in the asymmetric unit, corresponding to a solvent content of 38%. The structure was refined to 2.23 Å resolution with an *R*_cryst_ of 17.7% and an *R*_free_ of 22% (Tables 3 ▸ and 4 ▸). No electron density could be attributed to the residues of the N-terminal tag, which are consequently missing from the final model. The structure has no Ramachandran outliers, with 98% and 2% of the residues in the preferred and allowed regions, respectively.

#### 3.2.1. Overall structure   

**Figure 2:**
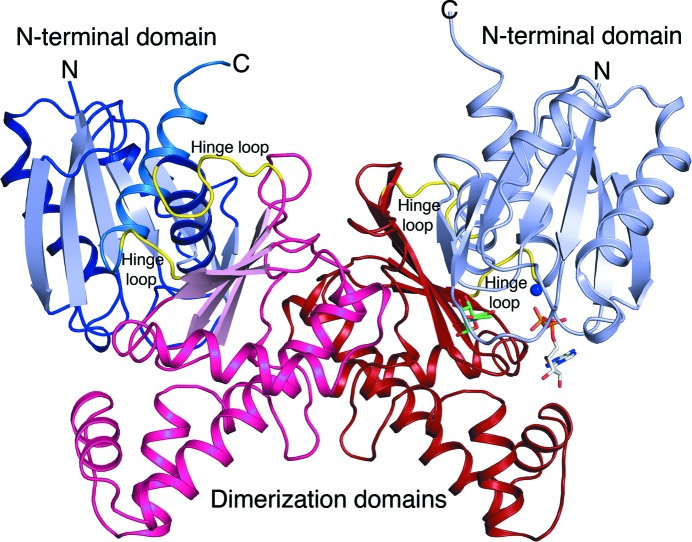
Overall structure of *F. nucleatum* apo *N*-acetylmannosamine kinase. The N-terminal domain is coloured in blue shades, the C-terminal dimerization domain in red shades and the the hinge loops are depicted in yellow. For clarity, based on the human hMNK structure (PDB entry 2yhy), ManNAc (green sticks), ADP (white sticks) and Mg^2+^ (blue sphere) have been modelled in the putative active site.

**Figure 3:**
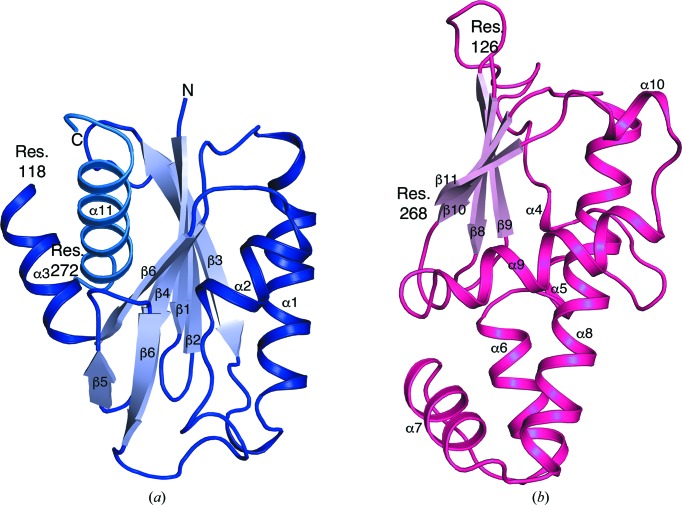
Overall structures of the N-terminal domain (*a*) and C-terminal dimerization domain (*b*). The helices and strands are numbered. The residues that span and flank each domain are marked. Domain 1 starts from the N-terminus and ends at residue 118 and then continues from residue 272 to the C-terminus (blue). Residues 126–268 form the dimerization domain.

FnNanK is a butterfly-shaped homodimer, as seen in other members of the ROK family (Fig. 2 ▸). The monomer structure has an elongated shape and is composed of two α/β domains that are connected by two hinge loops (residues 119–125 and 269–271). The putative active site is located in a large cleft between the N-terminal domain, which is made of two fragments (residues 1–118 and 272–291), and the slightly smaller C-terminal dimerization domain (residues 126–268). The N-terminal domain contains a central mixed, twisted five-stranded β-sheet (β1–β4 and β7) surrounded by four α-helices (α1–α3 and α11) and a short β-hairpin (β5–β6) (Fig. 3 ▸ *a*). The C-terminal dimerization domain consists of a mixed, twisted four-stranded β-sheet (β8–β11) that is sandwiched between the N-terminal domain and a cluster of α-helices and 3_10_-helices of the C-terminal domain (Fig. 3 ▸ *b*). Forty residues of the helix cluster and connecting loops of the C-terminal domain create a 1479 Å^2^ dimer interface stabilized by direct hydrogen bonds and solvent-mediated hydrogen bonds.

#### 3.2.2. The putative active site   

**Figure 4:**
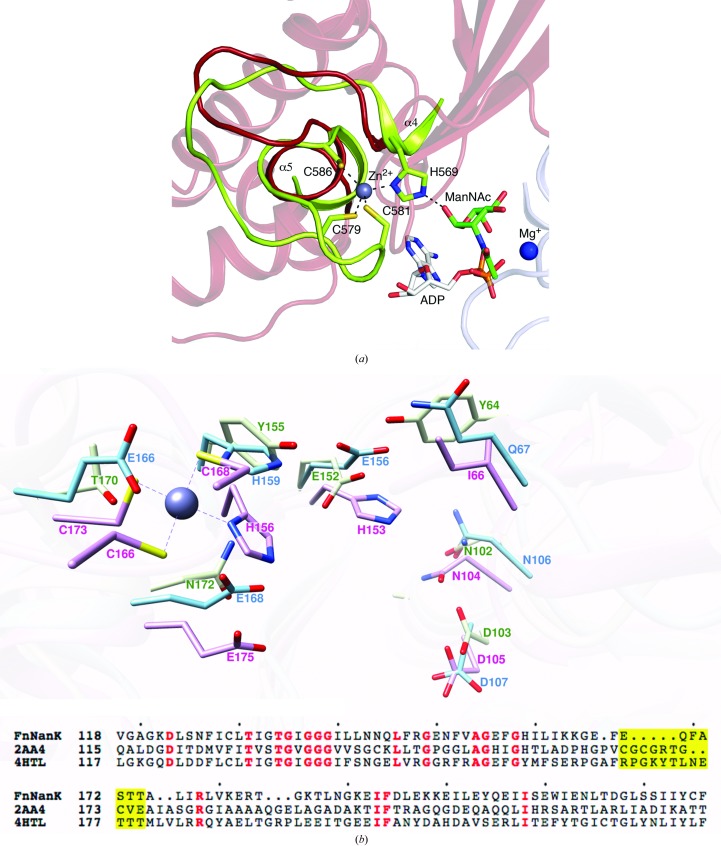
FnNanK lacks the cysteine-rich zinc-binding motif. (*a*) Structural comparison of apo FnNanK in red and substrate-bound (ManNAc, ADP and Mg^2+^) hMNK in green. (*b*) Superimposition of the substrate-binding regions of bacterial NanKs. The putative residues involved in catalysis in the substrate-binding site in FnNanK (blue) are superimposable with the corresponding residues in NanK from *E. coli* (EcNanK; PDB entry 2aa4, pink) and *L. monocytogenes* (LmNanK; PDB entry 4htl, green). The zinc-binding motif is only visible in EcNanK, which is represented by the coordination of Cys173, Cys166, Cys168 and His156 to the Zn atom (grey). The highly conserved histidine that coordinates ManNAc is present in FnNanK and EcNanK but corresponds to a tyrosine in LmNanK.

In this study, we report an apo structure of FnNanK. The *N*-acetylmannosamine kinase domain (hMNK) of the human bifunctional UDP-*N*-acetyl­glucosamine 2-epimerase/*N*-acetylmannosamine kinase shares 23% sequence identity with FnNanK and has been characterized both functionally and structurally (Martinez *et al.*, 2012 ▸). The structure of hMNK in complex with ManNAc and ADP (PDB entry 2yhy) can be superimposed on NanK with an r.m.s. deviation of 2.5 Å for 280 Cα atoms, making it possible to model the binding of ManNAc and ADP in the active site of FnNanK (Fig. 4 ▸ *a*). Residues that are involved in substrate and ATP binding are located in both the N-terminal and C-terminal domains. The conserved residues in hMNK (Asn516, Asp517, Arg477, Glu566, His569 and Glu588) that are required for the coordination of ManNAc (Martinez *et al.*, 2012 ▸) are superimposable with Asn106, Asp107, Gln67, Glu156, His159 and Glu168 in FnNanK.

The bacterial EcNanK (PDB entry 2aa4) and LmNanK structures (PDB entry 4htl) were superimposed with FnNanK. The r.m.s. deviations for the structural alignments of EcNanK (289 C^α^ atoms) and LmNanK (280 Cα atoms) with FnNanK are 2.6 and 1.9 Å, respectively. The putative active-site residues in FnNanK (Asn106, Asp107, Gln67, Glu156, His159 and Glu168) that are predicted to be involved in substrate binding are superimposable with those in EcNanK (Asn104, Asp105, Ile66, His153, His156 and Glu175) and LmNanK (Asn102, Asp103, Tyr64, Glu152, Tyr155 and Asn172) (Fig. 4 ▸ *b*).

#### 3.2.3. FnNanK lacks a zinc-binding site   

The common signature motifs of the ROK scaffold are (i) an N-terminal region containing the nucleotide-binding site with a D*x*G*x*T sequence motif, (ii) a strictly conserved catalytic aspartate within the active-site loop, (iii) an E*x*GH motif that interacts with the sugar substrate and (iv) a cysteine-rich zinc-binding motif with sequence C*x*CG*xx*GC*x*(E/D) (Conejo *et al.*, 2010 ▸). The first three signature motifs are also conserved in FnNanK.

Although FnNanK retains most of the consensus motifs unique to the ROK family, the lack of a zinc-binding site with sequence *x*CG*xx*GC*x*(E/D) is evident both in the sequence and structure alignments. The zinc-binding motif is implicated in upholding the structural integrity of the active site (Mesak *et al.*, 2004 ▸; Martinez *et al.*, 2012 ▸). A recent report suggested that mutation of the cysteines in the zinc-binding motif through site-directed mutagenesis renders *Bacillus subtilis* glucokinase inactive (Mesak *et al.*, 2004 ▸). The Zn atom is coordinated by three thiols within the cysteine-rich motif and a fourth coordinating conserved histidine relating the zinc-motif region to the substrate-binding site (Schiefner *et al.*, 2005 ▸). Superimposition of the residues that are involved both in zinc binding and substrate binding in PDB entries 2aa4 (pink) and 4htl (green) and in FnNanK (blue) highlights the absence of the cysteine-rich region in FnNanK (Fig. 4 ▸ *b*).

FnNanK lacks the zinc-binding motif, and sequence analysis of the known *N*-acetylmannosamine kinases shows that the consensus sequence *x*CG*xx*GC*x*(E/D) which denotes the zinc-motif region is not present in FnNanK. In LmNanK (PDB entry 4htl; Fig. 4 ▸ *b*) there seems to be no deletion; however, the loop contains no cysteine residues. The lack of zinc-binding sequence also extends to methicillin-resistant *Staphylococcus aureus* (MRSA) NanK (North *et al.*, 2013 ▸). However, FnNanK retains the highly conserved His159. The corresponding residue is His156 in EcNanK, and this histidine has been shown to bind both to the zinc ion and to ManNAc in human NanK (His569; Nocek *et al.*, 2011 ▸; Martinez *et al.*, 2012 ▸). Mutations of the two cysteine residues associated with zinc binding to serine and alanine in the *E. coli* Mlc repressor compromised its repressor function.

The three cysteine residues and histidine residue engaged in zinc-ion coordination are considered to be a distinct motif in the ROK family (Schiefner *et al.*, 2005 ▸). Although the three cysteines are not present in FnNanK, His159 is noted to be significantly shifted on superimposition with EcNanK. The measured distance between His159 in FnNanK and the substrate ManNAc in hMNK is twice as far compared with the distance between His569 in hMNK and ManNAc. In FnNanK, the glutamate residue Glu166 is markedly visible in place of the cysteine residues (Fig. 4 ▸ *b*). The structure of FnNanK in complex with substrate analogues and ATP (or an analogue) is required to predict the change in conformation that is needed to complete the binding of the substrate and ATP.

## 4. Concluding remarks   

In this paper, we present the crystal structure of apo FnNanK. In addition, we analyze and compare the sequence and structure of FnNanK with those of other *N*-acetyl­mannosamine kinases that display consensus features of the ROK superfamily. One of these signature motifs is the zinc-binding site, which is reportedly crucial in maintaining the structural integrity of the active site. We find that despite the absence of a zinc-binding motif in FnNanK, the major structural features that are implicated in enzymatic function are not compromised.

## Figures and Tables

**Table 1:** *F. nucleatum* NanK production information

Source organism	*F. nucleatum*
DNA source	Synthetic gene
Forward primer	CAAAAAAGCAGGCTTCATGAATATTTTAGCAATAGAT
Reverse primer	CAAGAAAGCTGGGTTTTATCTTTTATTAATTTTCTCT
Cloning vector	pMK vector
Expression vector	Gateway vector pET300 NT/DEST containing a sequence encoding an N-­terminal His_6_ tag
Expression host	*E. coli* BL21(DE3)
Complete amino-acid sequence of the construct produced	MHHHHHHITSLYKKAGFMNILAIDIGGTMIKYGLVSFDGKILSTDKIKTEASKGLNNILNKID-NIFKRYKENNPVGIAVSGTGQINGMIGKVIGGNPIIPNWIGTNLVKILEEKYNLPIVLENDVNCVALGEKWVGAGKDLSNFICLTIGTGIGGGILLNNQLFRGENFVAGEFGHILIKKGEFEQFASTTALIRLVKERTGKTLNGKEIFDLEKKEILEYQEIISEWIENLTDGLSSIIYCFNPANIILGGGVIEQGEPLINRIKNSLFKKIGPQFKEKLNITQAKLGNNAGMIGASYLLLEKINKR

**Table 2:** Crystallization of *F. nucleatum* NanK

Method	Vapour diffusion, sitting drop
Plate type	96-well Swissci plates
Temperature (K)	293
Protein concentration (mg ml^−1^)	14
Buffer composition of protein solution	20 m*M* Tris–HCl pH 8.0, 300 m*M* NaCl, 5% glycerol, 1 m*M* DTT
Composition of reservoir solution	0.2 *M* lithium sulfate monohydrate, 0.1 *M* Tris–HCl pH 8.5, 30%(*w*/*v*) PEG 4000
Volume of drop (nl)	200
Volume of reservoir (µl)	80

**Table 3:** Data collection and processing for *F. nucleatum* NanK Values in parentheses are for the outer shell.

Diffraction source	MAX-lab synchrotron
Wavelength (Å)	1.0
Temperature (K)	100
Detector	MAR CCD
Crystal-to-detector distance (mm)	210.69
Rotation range per image (°)	0.50
Total rotation range (°)	125.50
Exposure time per image (s)	30
Space group	*P*3_2_21
*a*, *b*, *c* (Å)	126.5, 126.5, 108.8
α, β, γ (°)	90, 90, 120
Mosaicity (°)	0.55
Resolution range (Å)	48.94–2.23 (2.31–2.23)
Total No. of observations	5433 (31890)
No. of unique reflections	49344 (4891)
Completeness (%)	100 (100)
CC_1/2_	0.99 (0.59)
Multiplicity	6.4 (7.1)
〈*I*/σ(*I*)〉	11.7 (1.64)
*R*_p.i.m._	0.019 (0.399)
Overall *B* factor from Wilson plot (Å^2^)	29.4

**Table 4:** Structure solution and refinement for *F. nucleatum* NanK Values in parentheses are for the outer shell.

Resolution range (Å)	48.94–2.23 (2.31–2.23)
Completeness (%)	100
σ Cutoff	
No. of reflections, working set	49333 (4893)
No. of reflections, test set	2384 (210)
Final *R*_cryst_ (%)	17.7
Final *R*_free_ (%)	22.1
No. of non-H atoms	
Protein	4486
Water	479
Total	4965
R.m.s. deviations from ideal geometry	
Bonds (Å)	0.010
Angles (°)	1.09
Average *B* factors (Å^2^)	
Protein	36.00
Water	42.10
Ramachandran plot	
Most favoured (%)	98
Allowed (%)	2.4
